# ﻿Life history and secondary production of *Anomalocosmoecusilliesi* Marlier, 1962 (Trichoptera, Limnephilidae) in a small stream in the northern Ecuadorian Paramo

**DOI:** 10.3897/zookeys.1111.85576

**Published:** 2022-07-11

**Authors:** Blanca Ríos-Touma, Andrea C. Encalada, Narcís Prat

**Affiliations:** 1 Facultad de Ingenierías y Ciencias Aplicadas, Ingeniería Ambiental, Grupo de Investigación en Biodiversidad, Medio Ambiente y Salud (BIOMAS), Universidad de las Américas, Quito, Ecuador Universidad de las Américas Quito Ecuador; 2 Instituto Biosfera, Laboratorio de Ecología Acuática, Colegio de Ciencias Biológicas y Ambientales, Universidad San Francisco de Quito, Quito, Ecuador Universidad San Francisco de Quito Quito Ecuador; 3 Grupo de Investigación FEHM (Freshwater Ecology Hydrology and Management), Departamento de Biología, Evolutiva, Ecología y Ciencias Ambientales, Facultad de Biología, Universidad de Barcelona, Barcelona, Spain Universidad de Barcelona Barcelona Spain

**Keywords:** Andean Caddisflies, Limnephilidae, secondary production

## Abstract

Life history of benthic faunas of tropical high-altitude cold environments are poorly studied. Here, monthly larval and adult data are presented for *Anomalocosmoecusilliesi* at Saltana Stream in Ecuador. In cold conditions throughout the year (6 °C), this species showed an asynchronous and continuous production. Larval density showed two peaks in August and April. All five larval instars were present in most months. Using the size-frequency method an annual rate of secondary production per biomass of 4.8 was calculated. The measured biomass was 785 mg/m^2^.

## ﻿Introduction

*Anomalocosmoecusilliesi* (Marlier, 1962) is the only species of the caddisfly family Limnephilidae found in Ecuador (Ríos-Touma et al. 2017). This species only inhabits streams and rivers located above 2,900 m a.s.l. (Jacobsen and Brodersen 2008), where water temperatures can be cold throughout the year. For example, Espinosa et al. (2020) found this species in streams with temperatures ranging from 1.5 to 11 °C.

In general, little is known about the life history (e.g., generation times, turnover, and secondary production) of tropical high-altitude insects (Jacobsen 2008). The few studies performed at high altitudes in Ecuador have shown that multiple size classes co-occur often and asynchrony in life cycles (Turcotte and Harper 1982; Jacobsen 2008; Studholme et al. 2017). In fact, *A.illiesi* larvae were studied by Turcotte and Harper (1982) in a small stream in southern Ecuador Paramo, where they found multiple size classes co-occurring throughout the year.

This species can be the dominant Trichoptera in some high-altitude streams in Ecuador. With this note on the life history and secondary production of *A.illiesi*, we aim to contribute to the knowledge of high-altitude tropical aquatic insects that remain understudied in taxonomy, ecology, and physiology.

## ﻿Materials and methods

### ﻿Study site

We conducted this study at the Saltana Creek (0°19'1.80"S, 78°13'8.8"W), a first-order stream of Esmeraldas River Basin that flows to the Pacific Ocean in Ecuador. This stream is located at 3,800 m a.s.l. The catchment area is covered by mixed Paramo vegetation and is protected in the Paluguillo Hydrological Protection Area. We visited the stream monthly from July 2009 to June 2010. Discharge was higher from June to August, while base flow conditions were found September–March. Temperature ranged from 5.5 to 10.6 °C, conductivity from 63 to 70 μS/cm, and pH from 6.5 to 7.8. Oxygen was close to saturation during the entire study period. More information about this stream can be found in Holzenthal and Ríos-Touma (2012) and Vimos et al. (2015).

### ﻿Larval and adult sampling

*Anomalocosmoecusilliesi* larvae were obtained through 12 randomly taken monthly benthic Hess samples with an area of 0.02 m^2^ and mesh of 250 µm. Also, a 2-minute kick-sample was collected every month covering all the habitats, including shoreline and aquatic vegetation. We fixed each sample with 5% formalin and preserved it in 90% ethanol. Head capsule width (**HCW**) and body length (**BL**) of all specimens were recorded with an Olympus SZX 16 stereomicroscope calibrated for measuring with an ocular micrometer. The HCW was used since it is an accurate measurement to produce histograms and determine size classes (Komzák and Sedlák 2002; Brand and Miserendino 2012). Twenty random larvae of all size classes determined by the HCW were selected to determine dry mass and the relationship between body length and biomass. We also assessed the correlation between HCW and body length.

Adults were sampled using three amphibious emergence traps (Megaview Science, model BD5740A, Taiwan) (1.1 × 1.1 × 1.1 m) that were placed immediately above the stream covering the entire stream width from one side to the other and were operated for 24 h each month. Additionally, 12 flight-intercept traps (vertical, across the stream) and eight platform sticky traps (horizontal, placed above water level) were sampled monthly for 24 h. We used Tree Tanglefoot sticky compound on the acetate sheets (210 × 297 mm) of the traps and citric-based solvent to remove the specimens from the traps (following Encalada and Peckarsky 2007). All collected specimens were preserved in 96% ethanol.

### ﻿Secondary production

We calculated secondary production following the size frequency method (Hamilton and Hynes 1969; Benke 1979; Benke and Huryn 2007). This non-cohort method assumes that mean size distribution from samples collected throughout a year is similar to a mortality curve for an average cohort (see details in Benke and Huryn 2007). We were able to catch a female and kept her alive in a vial with river water until she expelled the eggs into the water. We then refrigerated the eggs in the river water to 6 °C (similar to stream temperature) with 12 h of light. It took 12 weeks for the eggs to hatch. Unfortunately, we were not able to keep the larvae alive under laboratory conditions, but with this data we assumed a cohort production interval of six months, considering the constant cold temperatures of the stream. Our assumption of six months comes from our hatching data (three months for the egg to hatch in lab conditions), and from other studies in Limnephilids under similar temperatures (Gislason and Sigfusson 1987), where larval development and pupation took approximately 3–4 months, and where adults only lived a few days.

## ﻿Results

### ﻿Month larval density

**Figure 1.**
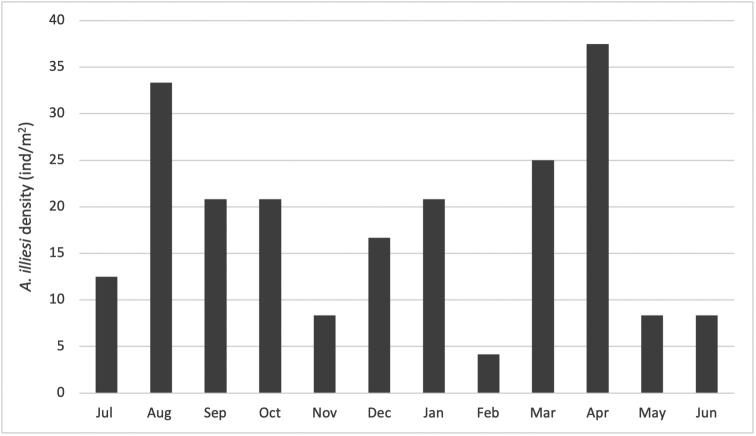
*Anomalocosmoecusilliesi* total monthly larval density (ind/m^2^) at Saltana stream, Ecuador from July 2009 to June 2010.

Density ranged from 4.2 ind/m^2^ in February to 37.5 ind/m^2^ in April. Larvae were present in all months (Fig. 1).

**Figure 2.**
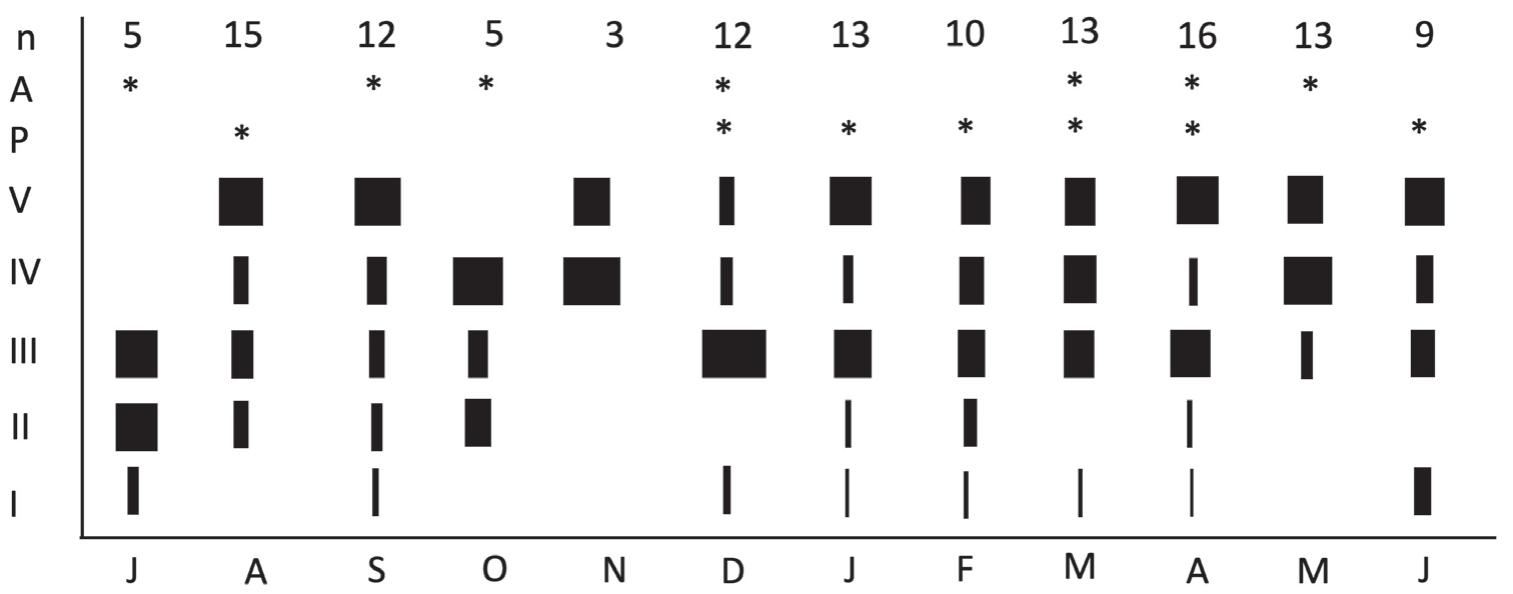
Size frequency of *Anomalocosmoecusilliesi* larval instars from July 2009 to June 2010 at Saltana Stream, Ecuador. The presence of pupae (P) and adults (A) are marked with asterisks, and the number of larvae taken into consideration (n) is marked for each month.

Using the HCW, we were able to separate the five larval instars (Fig. 2). All instars were found in most months, showing an asynchronous and continuous pattern throughout the year. Pupae were found in August (3), December (1), January (1), February (1), March (2), and June (1).

### ﻿Adults

Adults were found on our sticky traps in July (1), September (2), October (2), and December (1) of 2009 and from March to May in 2010 (3, one each month). Only four individuals were found in our emergence traps, three in September 2009 and one in May 2010.

### ﻿Secondary production

Head capsule width (HCW) and body length (L) were highly correlated (R^2^ = 0.95). With the weighed larvae and body length we calculated the biomass of the five instars in our monthly samples with the following equation: W = 0.0072L^2.5615^.

Our data showed an annual biomass of 785 mg/m^2^ (Table 1), with an annual production per biomass rate (P/B) of 4.8, considering all the instars and larvae found throughout the year and a Cohort P/B of 2.4, assuming two cohorts a year.

**Table 1.** Annual production of *Anomalocosmoecusilliesi* using the size frequency method, for monthly larval samples from Saltana stream, Ecuador. No. lost = individuals lost between instars; Ŵ = mean individual mass between two instars; biomass lost = biomass lost between instars. Production (P) is the result of summing the biomass lost between each instar per the number of instars (stages, or size classes), calculated according to Benke and Huryn (2007). P/B is the production per biomass rate. CPI is the cohort production interval, which for *A.illiesi* we assumed was 6 months.

Instar	Density	Individual mass	(No./m^2^)	Biomass (mg/m^2^)	Mass at loss (mg)	Biomass lost (mg/m^2^)	Times no. of size classes (instars)
	no./m^2^	W (mg)	ΔN	N × W	Ŵ = (W1 +W2)/2	ŴΔN	ŴΔN × 5
1	12.50	0.08	-70.83	1.00		-10.75	-53.73
					0.15		
2	83.33	0.22	-104.17	18.61		-52.50	-262.48
					0.50		
3	187.50	0.78	20.83	147.12		27.32	136.61
					1.31		
4	166.67	1.84	41.67	306.46		90.32	451.58
					2.17		
5	125.00	2.50	125.00	312.01		312.01	1560.03
					2.50		
			Σ Biomass:	785.19		Production (uncorrected)	1885.73
			P/B Cohort	2.40		Annual P	3771.47
			Annual P/B	4.80		**CPI = 2**	

## ﻿Discussion

Continuous production and asynchronous life cycles have been previously reported for tropical taxa (Turcotte and Harper 1982; Jacobsen 2008; Studholme et al. 2017). However, data on tropical high-altitude cold streams are scarce. We assumed two generations a year based on hatching times from a single egg mass, the constant cold temperature of the stream, and information on other cold water-affiliated Limnephilidae species from Europe. This, however, is based on a single hatching observation and further studies must be done to test this assumption. Unfortunately, despite our efforts to maintain constant cold conditions in laboratory conditions, we were unable to keep additional larvae alive until emergence. Larvae in very cold environments, like in the boreal and subalpine zones in Sweden, showed similar patterns to those of milder climates, with growing periods when the temperatures were between 4 and 13 °C, but some of them took 1–2 years to reach emergence (Ulfstrand 1968). The limnephilid *Apataniazonella* Zetterstedt, 1840 showed one generation per year in Iceland even though the spring-fed stream temperatures did not fluctuate much during the year (3–8 °C in summer and -1–4 °C in winter; Gislason and Sigfusson 1987). We believe that growth rates could be similarly slow for *A.illiesi*, but with continuous emergence.

The annual P/B found corresponds to the most frequent values worldwide (below 6), provided by Benke (1993), and is similar to other caddisflies from other parts of the world. Annual production and Annual P/B was in the same range found for caddisflies in Patagonia, and Ecuadorian Paramos (Brand and Miserendino 2012; Studholme et al. 2017). Jacobsen (2008) predicted that because of low temperatures and oxygen availability, production in Paramo stream insects will be lower compared to its lowland counterparts. We found values of annual production that are similar to the most productive tropical Trichoptera (Ramirez and Pringle 1998) including *Helicopsyche* in the southern Ecuadorian Paramo streams Studholme et al.(2017). To our knowledge, this is the first report of secondary production of a Limnephilid in South America.

Larval density could be related to flow, with lower densities at July and February, when spates occurred in the stream (Holzenthal and Ríos-Touma 2012; Vimos et al. 2015). Hydrology and temperature (Prat 1981; Wagner and Schmidt 2004; Armitage 2006) can strongly affect community composition and therefore the annual production of caddisflies. For example, Prat (1981) showed that lower temperatures due to hydrological changes in a reservoir in Spain caused a slower growth rate for *Psychomylapusilla* Fabricius,1781 and the production of a single generation, compared to two generations in the previous year. Wagner and Schmidt (2004) found that community diversity declined in years with random flows with effects also seen in the emergence of Ephemeroptera, Plecoptera and Trichoptera taxa. Also, work on other Paramo caddisflies has shown that, besides temperature, food availability such as periphyton density results in secondary production differences among streams with similar temperatures (Studholme et al. 2017). In our stream temperature was constant through the year and it was independent from hydrology. Therefore, we conclude that a main factor controlling density of this and other macroinvertebrates will be unpredictable spates that can occur in these as well as in other streams around the world (Wagner and Schmidt 2004; Ríos-Touma et al. 2011; Tonkin et al. 2017).
